# Supplementary material for: PreDicta chip-based high resolution diagnosis of rhinovirus-induced wheeze
Source: Nat Commun. 2018 Jun 18;9:2382. doi: 10.1038/s41467-018-04591-0 (PMC6006174; doi:10.1038/s41467-018-04591-0)
Supplement: Supplementary file 1 — Supplementary Information [file 41467_2018_4591_MOESM1_ESM.pdf]

PreDicta chip-based high resolution diagnosis of rhinovirus-induced wheeze

Niespodziana *et al.*

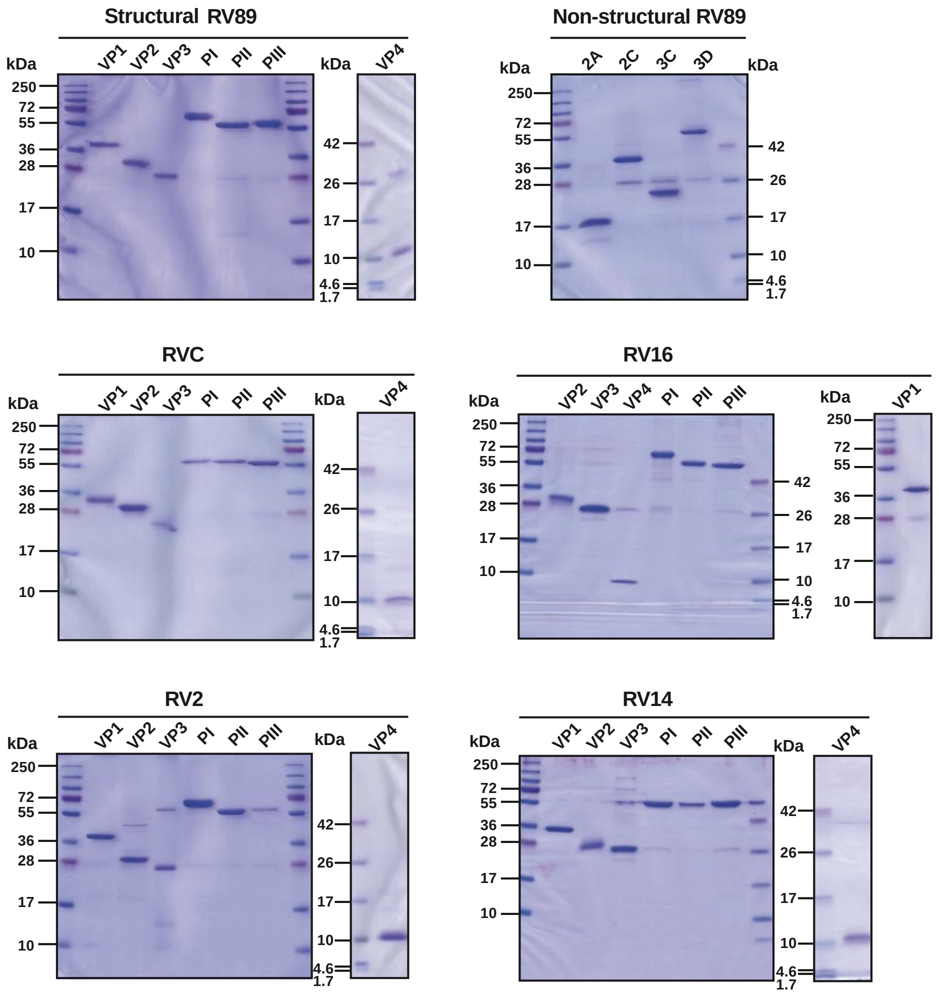

Supplementary Fig. 1. Purification of recombinant RV structural and non-structural proteins. Coomassie-stained SDS-PAGE containing recombinant capsid proteins from RV89, RV-C, RV16, RV2, and RV14 (VP1, VP2, VP3, VP4), recombinant VP1 fragments thereof (PI, PII, PIII) and non-structural proteins from RV89 (2A, 2C, 3C, 3D). Molecular masses in kilo Dalton (kDa) are indicated on the left margins.

a

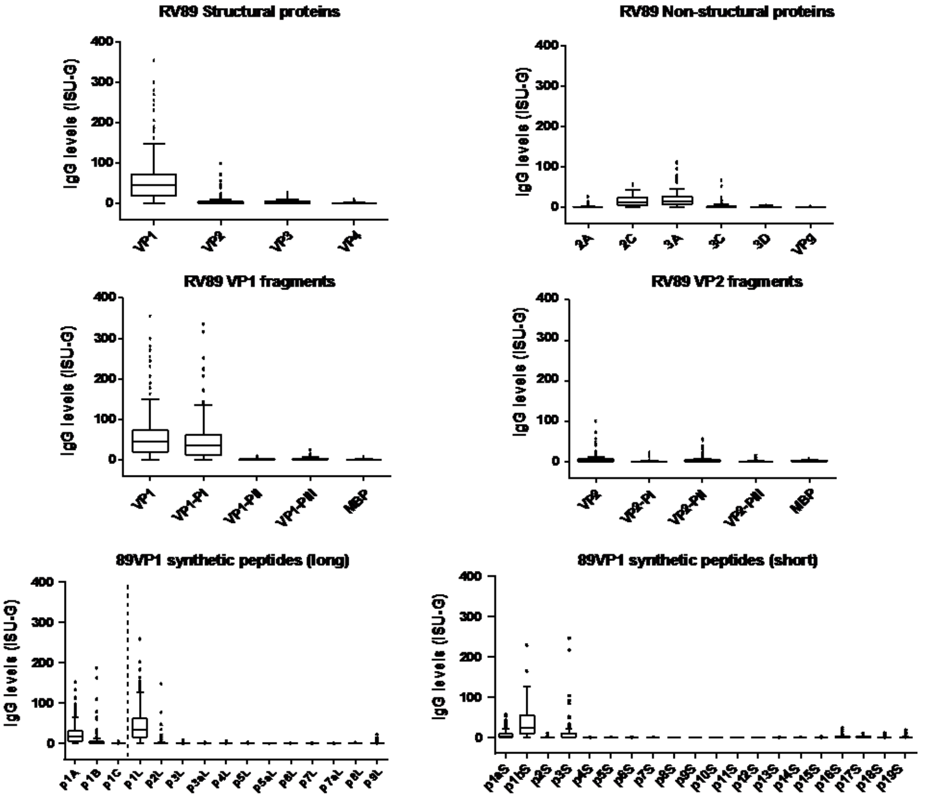

b

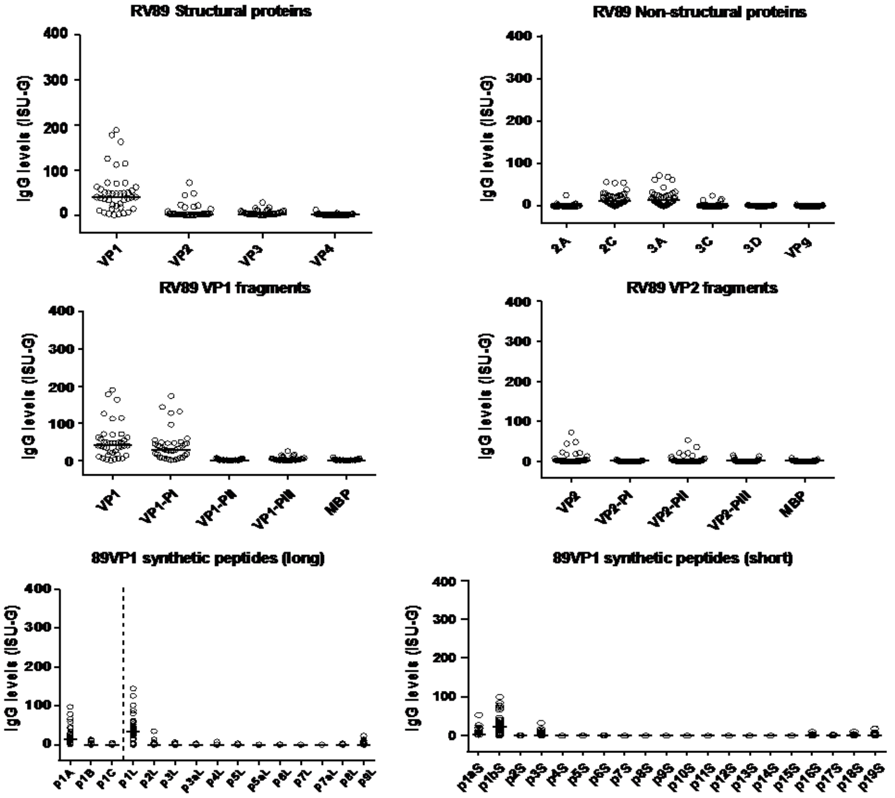

Supplementary Fig. 2. IgG antibody responses to recombinant RV89 proteins and RV-derived peptides in sera from children taken during the acute wheezing attack. IgG levels (ISU-G, y-axes) to recombinant RV89 structural (VP1-VP4) (top left), non-structural proteins (2A, 2C, 3A, 3C, 3D, VPg) (top right), VP1 and VP2 fragments (PI-PIII), and maltose binding protein (MBP) (middle panels) and VP1-derived long and short peptides (bottom panels) measured in sera of (***a***) 120 children and (***b***) in sera of 41 RVA-positive children are shown as box plots and scatter plots, respectively. Fifty % of the values are within the boxes, outliers are indicated by dots. Horizontal lines within boxes and plots indicate median values.

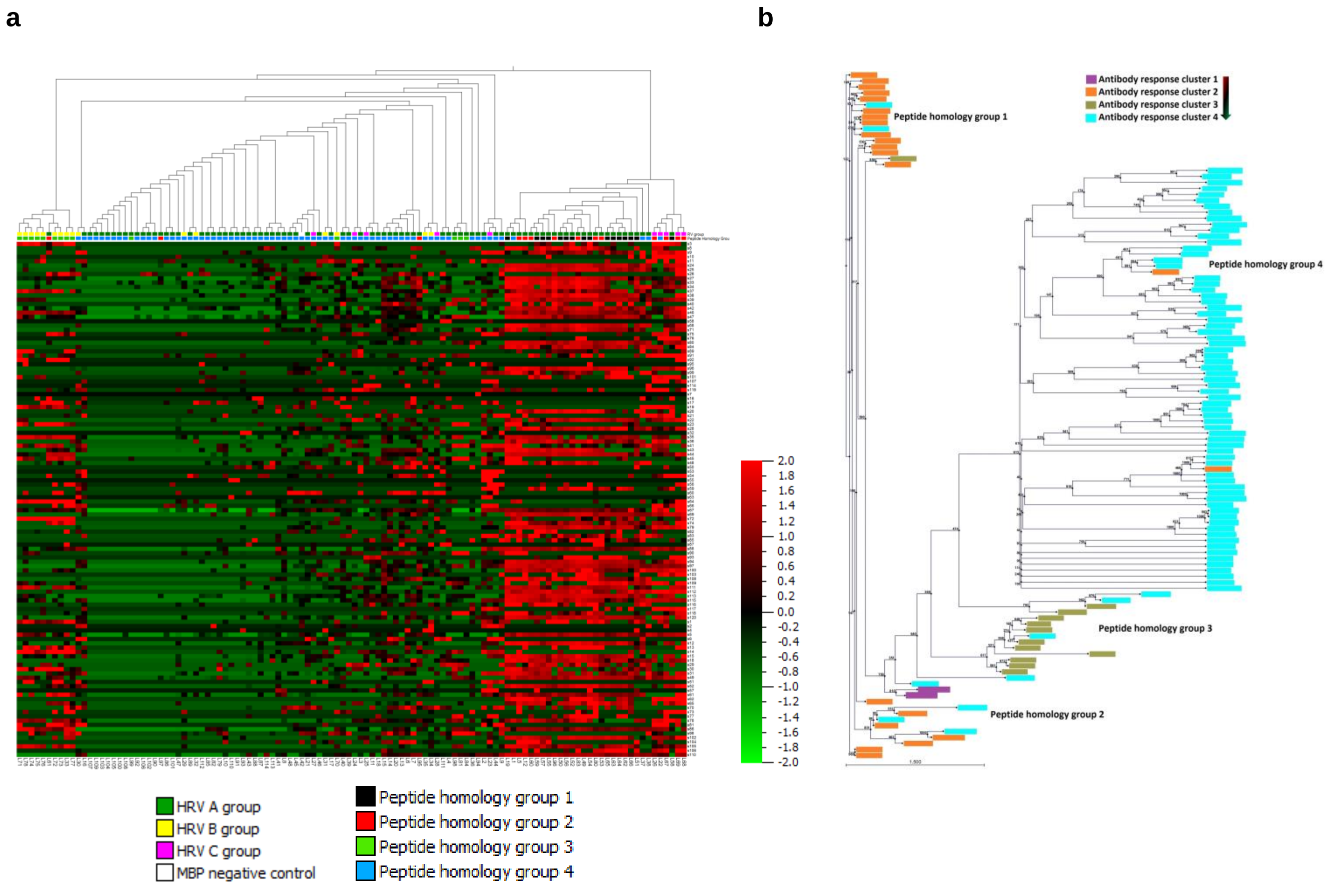

Supplementary Fig. 3. Heatmap representation of RV-specific antibody responses measured by RV-chip. (***a***) Shown are IgG levels (ISU-G) to micro-arrayed RV components (bottom line) measured in serum samples obtained from 120 children during the acute wheezing attack according to age (top to bottom: youngest to oldest). IgG levels are shown by colour codes where red represents high and green low values, normalized to have a mean of 0 and a variance of 1. Rows depict specific antibody responses for each subject and columns depict antigen-specific antibody responses throughout all subjects. The color-coded annotations on top of the heat map represent pre-determined groups of peptides that refer either to the RV group or the peptide homology groups as determined in *B*. (***b***) Homology distance cladogram of the micro-arrayed RV components built using the Neighbor Joining algorithm and 1000 bootstrap replicates. The amino acid sequences of the peptides were aligned with a Gap open cost of 10.0 and a Gap extension cost of 1.0. The peptides were then color-coded based on the antibody response cluster that they belong to, with purple representing the cluster with the highest mean measurement and light blue the cluster with the lowest mean measurement and orange and green clusters in between. The antibody response clusters were determined using unsupervised K-means clustering (K=4).

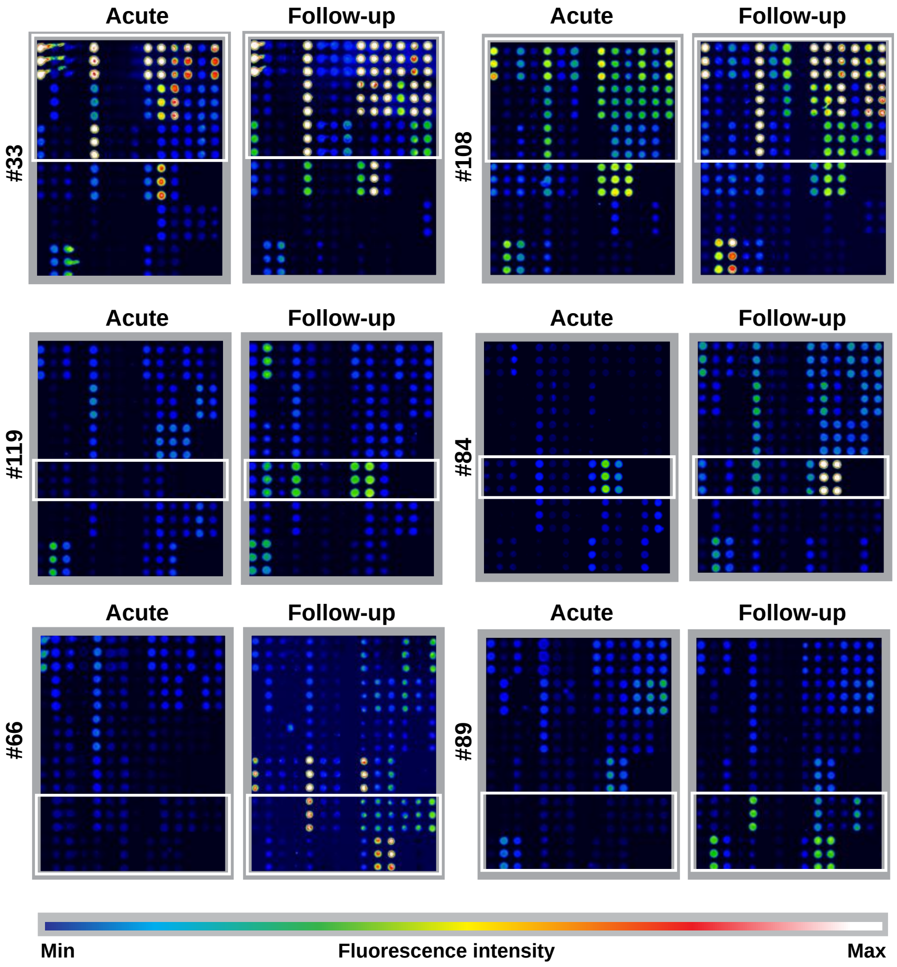

Supplementary Fig. 4. Representative alterations of IgG antibody responses to micro-arrayed RV proteins, VP1 protein fragments and VP1 N-terminal peptides after RV infection. Scan images from 6 representative patients (#33, #108, #119, #84, #66, #89) obtained by testing serum samples during and after the wheezing attack. Increasing fluorescence intensities from blue to red/white correspond to the amount of bound antibodies. RV proteins and peptides of RV-A, RV-C and RV-B species are boxed.

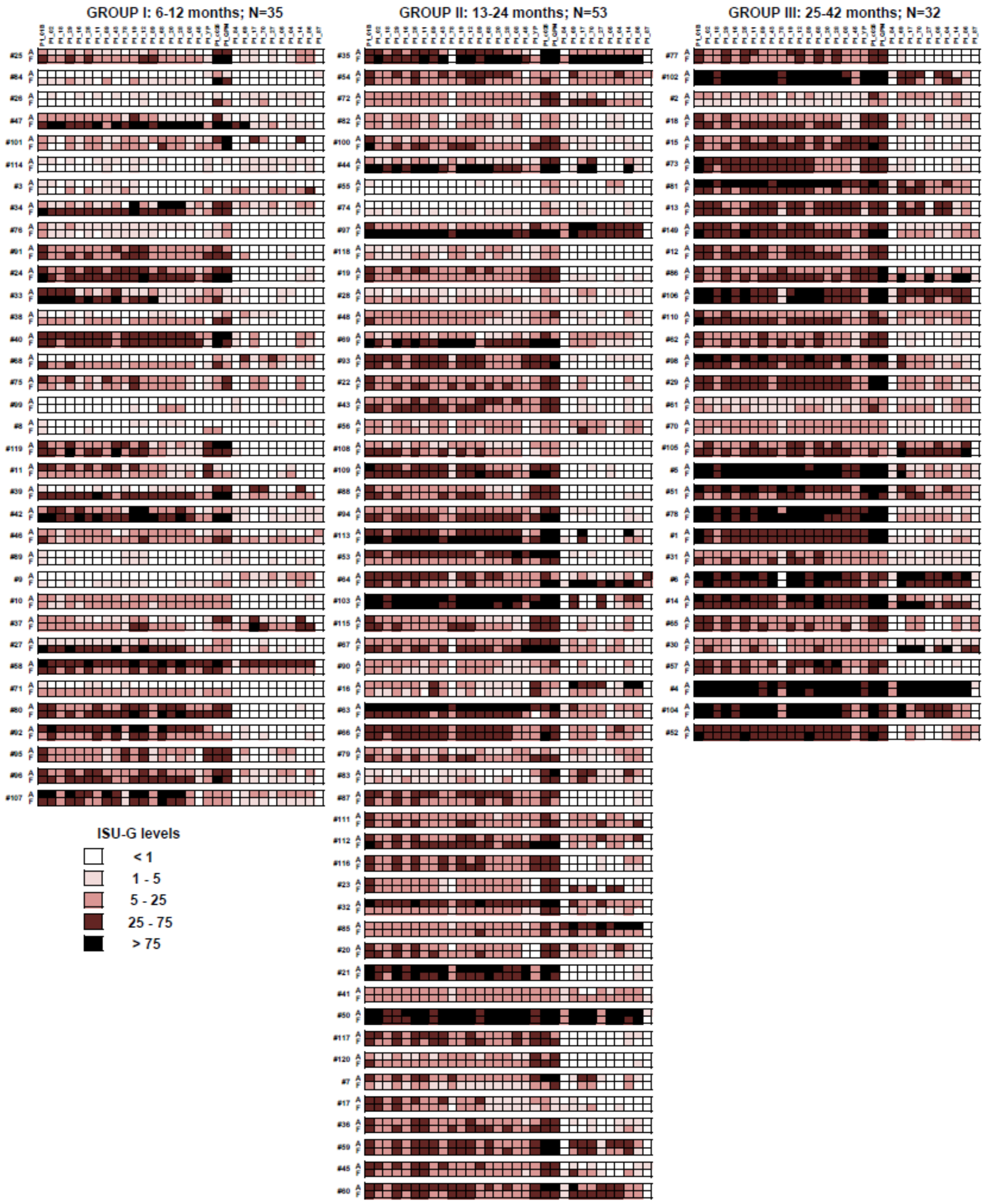

Supplementary Fig. 5. IgG levels to the N-terminal VP1 peptides in children according to age (see **Table 1**: groups I-III). Shown are IgG levels (color-coded according to ISU-G levels) to the N-terminal VP1 peptides from RV-A, RV-C and RV-A (top line) at the acute visit (A) and follow-up visit (F) for all children (left margins) in each age group.

Supplementary Table 1. Recombinant RV proteins, protein fragments and VP1 synthetic peptides spotted on the PreDicta chip.

Antigens	RV source strains	Recombinant protein/synthetic peptide	Total no. of antigens	Rationale
*Structural proteins, VP1-VP4	RV-A: 02, 16, 89<br>RV-C: YP<br>RV-B: 14	Recombinant proteins	20	To characterize RV-specific antibody responses to capsid proteins
*MBP fusion proteins, containing VP1 fragments	RV-A: 02, 16, 89<br>RV-C: YP<br>RV-B: 14	Recombinant proteins	15	To map epitopes of VP1
*MBP fusion proteins, containing VP2 fragments	RV-A: 89	Recombinant proteins	3	To map epitopes of VP2
*MBP fusion proteins, containing VP3 fragments	RV-A: 89	Recombinant proteins	3	To map epitopes of VP3
*Non-structural proteins	RV-A: 89	Recombinant proteins	6	To characterize RV-specific antibody responses to non-structural proteins
†VP1 N-terminal peptides	RV-A: 18 RV strains<br>RV-C: 3 RV strains<br>RV-B: 9 RV strains<br>HEV: 1 strain	Synthetic peptides	31	Detection of species- and strain-specific responses
‡VP1 overlapping peptides	RV-A: 89	Synthetic peptides	20	To map epitopes of VP1
‡VP1 non-overlapping peptides	RV-A: 89	Synthetic peptides	16	To map epitopes of VP1
§Allergens	n.a.	Recombinant or natural proteins	16	Calibration of IgG and IgA responses
§Maltose Binding Protein	n.a.	Recombinant protein	1	Control proteins for the MBP fusion proteins

Definition of abbreviations: n.a., not applicable.
*Supplementary Table 5.
† Supplementary Tables 2 and 3.
‡ Supplementary Table 4.
§ Supplementary Table 6.

Supplementary Table 2. Clustering and re-clustering of VP1 peptides according to sequence identities and chemical properties.

Cluster	Peptides according to sequence identities (≤ 5 aa)	Re-cluster	Peptides according to sequence identities and chemical properties	Peptide selected for CHIP	RV strain
**A 1**	1A, 1B, 2, 9, 10, 15, 18, 21, 24, 29, 31, 32, 33, 34, 38, 39, 40, 47, 49, 50, 74, 81, 85, 90, 98	**A I**	1A, 1B, 15, 74	**01p1**	**1B**
**A 2**	23, 2, 16, 21, 25, 29, 30, 40, 41, 49, 54, 61, 62, 73, 81, 82, 85, 96	**A II**	2, 9, 21, 23, 24, 30, 32, 33, 34, 49, 50, 57, 76, 90	**02p1**	**2**
**A 3**	7, 36, 58, 88, 89	**A III**	7, 36, 58, 88, 89	**89p1**	**89**
**A 4**	8, 95	**A IV**	8, 95	**08p1**	**8**
**A 5**	44, 10, 15, 21, 24, 25, 29, 31, 40, 47, 54, 56, 62, 66, 74, 77, 81, 82, 85, 90, 98, 100	**A V**	10, 25, 62, 77, 82	**25p1**	**25**
**A 6**	11, 34, 50, 57, 60, 67, 75	**A VI**	11, 38, 60, 66, 100	**11p1**	**11**
**A 7**	12, 73	**A VII**	12, 73	**12p1**	**12**
**A 8**	13, 15, 16, 34, 40, 41, 50, 54, 55, 56, 61, 73, 74, 81, 85, 96, 98	**A VIII**	13, 16, 40, 41, 54, 55, 56, 61, 67, 73, 96	**16p1**	**16**
**A 9**	19, 22, 56, 64, 82, 94	**A IX**	18	**18p1**	**18**
**A 10**	20, 53, 68	**A X**	19, 22, 64, 82, 94	**19p1**	**19**
**A 11**	28, 51, 53, 68, 103	**A XI**	20	**20p1**	**20**
**A 12**	76, 33, 34, 43, 50, 57, 75	**A XII**	28, 46, 51, 53, 65, 71, 80, 103	**28p1**	**28**
**A 13**	43, 75, 76	**A XIII**	29, 31, 44, 47, 81, 85, 98	**29p1**	**29**
**A 14**	45	**A XIV**	43, 75	**43p1**	**43**
**A 15**	59, 39, 40, 56, 63	**A XV**	45	**45p1**	**45**
**A 16**	46, 51, 53, 65, 68, 71, 80, 103	**A XVI**	59, 39, 56, 63	**59p1**	**59**
**A 17**	78	**A XVII**	68	**68p1**	**68**
		**A XVIII**	78	**78p1**	**78**
**B 1**	3, 6, 14, 35, 37, 72, 79, 83, 92	**B I**	3, 6, 14, 35, 37, 72, 79, 83, 92	**14p1**	**14**
**B 2**	4	**B II**	4	**04p1**	**4**
**B 3**	5, 26, 42, 99	**B III**	5, 26, 42, 99	**05p1**	**5**
**B 4**	17, 37, 52, 84	**B IV**	17, 48, 52, 84	**17p1**	**17**
**B 5**	27, 93, 97	**B V**	27, 93, 97	**27p1**	**27**
**B 6**	48, 52, 70	**B VI**	69	**69p1**	**69**
**B 7**	69	**B VII**	70, 91	**70p1**	**70**
**B 8**	70, 91	**B VIII**	84	**84p1**	**84**
**B 9**	86	**B IX**	86	**86p1**	**86**
**C 1**	YP, c024	**C I**	YP, c024	**YPp1**	**YP**
**C 2**	QCE, c026, QPM	**C II**	QCE, c026, QPM	**QPMp1**	**QPM**
**C 3**	c025	**C III**	c025	**C025p1**	**c025**
**E 1**	68	**E I**	68	**68p1***	**68**

*68p1 belongs to Enterovirus;

Supplementary Table 3. Synthetic VP1 N-terminal peptides.

Peptide	Species	Strain	Amino acid sequence	Position*	Length [aa]†	Molecular weight [Dalton]	GenBank accession number
**01p1**	RV-A	1B	NPVENYIDEVLNEVLVVPNIKESHHTTSNSAPLLDAAETG	1-40	40	4331	AAQ19856
**02p1**	RV-A	2	NPVENYIDEVLNEVLVVPNINSSNPTTSNSAPALDAAETG	1-40	40	4169	AAQ19857
**04p1**	RV-B	4	GLGEDLMEVIVDKTHQTLASVKSDSKHTQKVPALTANETG	1-40	40	4250	AAQ19859
**05p1**	RV-B	5	GLEDDLVEVIVDKAQQTLASIKSDSKHTQKVPSLTANETG	1-40	40	4267	AAQ19860
**08p1**	RV-A	8	NPIEQFTEAVLNEVLVVPNTQASNGSIANSAPALDAAETG	1-40	40	4053	ACK37369
**11p1**	RV-A	11	NPVEDYVDGILNEVLVVPNIKESQATTSNSAPALDAAETG	1-40	40	4143	AAQ19866
**12p1**	RV-A	12	NPVERYVDEVLNEVLVVPNINKSNGQLSNAAPALDAAETG	1-40	40	4222	AAQ19867
**14p1**	RV-B	14	GLGDELEEVIVEKTKQTVASISSGPKHTQKVPILTANETG	1-40	40	4206	AAQ19869
**16p1**	RV-A	16	NPVERYVDEVLNEVLVVPNINESHPTTSNAAPVLDAAETG	1-40	40	4275	AAQ19871
**17p1**	RV-B	17	GFEDELEEVVIDKMKQVTASSQSGPKHTQKVPALSANETG	1-40	40	4287	AAQ19872
**18p1**	RV-A	18	NPVENYIDEVLNEVLVVPNVNESHAITSNSAPALDAAETG	1-40	40	4207	AAQ19873
**19p1**	RV-A	19	NPVEKYVDTILNEVLVVPNINESHPSTSNAAPALDAAETG	1-40	40	4158	ACK37375
**20p1**	RV-A	20	NPVERYTEAILNEVLVVPNITSSNSQTSNAAPALDAAETG	1-40	40	4158	ACK37376
**25p1**	RV-A	25	NPIENYVDQVLNEVLVVPNIKESHPSTSNSAPILDAAETG	1-40	40	4276	ACK37379
**27p1**	RV-B	27	GLGEELEEVIVDKAKQTIASVSSNSKHTQKVPTLSASETG	1-40	40	4170	ACK37442
**28p1**	RV-A	28	NPVEKYTEALLNEVLVVPNINPSNAQTTNAAPALDAAETG	1-40	40	4151	AAQ19883
**29p1**	RV-A	29	NPVENYVDEVLNEVLVVPNIRESHPSTSNSAPILDAAETG	1-40	40	4291	ACK37381
**43p1**	RV-A	43	NPVENYVDEILNQVLVVPNTVESHSTTSNAAPALDAAETG	1-40	40	4181	ACK37387
**45p1**	RV-A	45	NPVEQFAEAVLDQVLVVPNTRPSDGLIANSAPALDAAETG	1-40	40	4091	ACK37388
**59p1**	RV-A	59	NPVENYVNDVLNEVLVVPNIQESHPTTSNAAPALDAAETG	1-40	40	4204	AAQ19914
**68p1**	RV-A	68	NPVEKYTEAVLNEVLVVPNIPASNTQTSNAAPALDAAETG	1-40	40	4110	ACK37406
**69p1**	RV-B	69	GLGEELEEVVIDKMKQVTASVQSGSKHTQKVPALSASETG	1-40	40	4170	ACK37407
**70p1**	RV-B	70	GFEGELEEVVIDKMKQVTASSQSGPKYTQKVPALSANETG	1-40	40	4255	AAQ19925
**78p1**	RV-A	78	NPVEEYVDQVLNEVLVVPNIKESKPQSSNSAPVLDAAETG	1-40	40	4281	ACK37439
**84p1**	RV-B	84	GLEDVLEEVIVDKAKQTIASINSNSKYTQQVPTLSASETG	1-40	40	4265	AAQ19939
**86p1**	RV-B	86	GLGDELEEVIVEKTKQTLASVATGSKYTQKVPSLSANETG	1-40	40	4180	ACK37420
**89p1**	RV-A	89	NPVENYIDSVLNEVLVVPNIQPSTSVSSHAA‡	1-31	31	3293	AAQ19944
**YPp1**	RV-C	C_YP	NPVEDYIDKVVDTVLQVPNTQPSGPQHSIQPSALGAMEIG	1-40	40	4246	YP_001552435
**QPMp1**	RV-C	C_QPM	NPVEEFVEHTLKEVLVVPDTQASGPVHTTKPQALGAVEIG	1-40	40	4238	ABP38410
**c025p1**	RV-C	C_025	NPVEQFVDNVLEEVLVVPNTQPSGPIHTTKPTALSAMEIG	1-40	40	4273	ABU62849
**68p1**	HEV	68	LDHLHGAEAAYQVESIIKTATDTVKSEIDAELGVVPSLNA	1-40	40	4207	AAQ19942

*Position denotes the corresponding region of the VP1 protein.
†Length: number of amino acids, aa.
‡Due to difficulties with peptide synthesis, P1A (Table E4) has been used for analysis.

Supplementary Table 4. 89VP1-derived peptides spotted on the PreDicta chip.

Peptide*	Amino acid sequence	Position‡	Lenght [aa]§	Molecular weight [Dalton]	Motifs‖
**p1A**	MNPVENYIDSVLNEVLVVPNIQPSTSVSSHAA	2-32	32	3424	
**p1B**	PALDAAETGHTSSVQPEDMIETRYVITDQTRDET	33-66	34	3778	
**p1C**	SIESFLGRSGCIAMIEFNTSSDKTEHDKIGKGFK	67-100	34	3734	
**p1aS**	NPVENYIDSVLNEVLVV	1-17	17	1916	
**p1bS**	NPVENYIDSVLNEVLVVPNIQ†	1-21	21	2368	
**p2S**	VVPNIQPSTSVSSHAAPALD†	16-35	20	1990	
**p3S**	APALDAAETGHTSSVQPEDM†	31-50	20	2027	
**p4S**	QPEDMIETRYVITDQTRDET†	46-65	20	2440	
**p5S**	TRDETSIESFLGRSGCIAMI†	61-80	20	2186	
**p6S**	CIAMIEFNTSSDKTEHDKIG†	76-95	20	2239	NIm-IA, NIm-IB
**p7S**	HDKIGKGFKTWKISLQEMAQ†	91-110	20	2345	
**p8S**	QEMAQIRRKYELFTYTRFDS†	106-125	20	2582	
**p9S**	TRFDSEITIVTAAAAQGDDS†	121-140	20	2068	NIm-IB
**p10S**	QGDDSGHIVLQFMYVPPGA†	136-154	19	2031	
**p11S**	VPPGAPVPEKRDDYTWQSGTN†	150-170	21	2312	ICAM-1
**p12S**	QSGTNASVFWQEGQPYPRFT†	166-185	20	2300	
**p13S**	YPRFTIPFMSIASAYYMFYD†	181-200	20	2483	
**p14S**	YMFYDGYDGDSAASKYGSVV†	196-215	20	2195	
**p15S**	YGSVVTNDMGTICVRIVTSN†	211-230	20	2129	ICAM-1
**p16S**	IVTSNQKHDLNIVCRIYHKA†	226-245	20	2352	
**p17S**	IYHKAKHIKAWCPRPPRAVA†	241-260	20	2342	
**p18S**	PRAVAYQHTHSTNYIPSNGE†	256-275	20	2242	
**p19S**	PSNGEATTQIKTRPDVFTVTNV	271-292	22	2375	NIm-IV
**p1L**	NPVENYIDSVLNEVLVVPNIQPSTSVSSHAAPAC**	1-33	34	3564	
**p2L**	LDAAETGHTSSVQPEDMIETRYVITDQTRDETSC**	34-66	34	3800	
**p3L**	IESFLGRSGCIAMIEFNTSSDKTEHDKIGKGFKTC**	67-102	35	3851	
**p3aL**	IESFLGRSGCIAMIEFNTSSDKTEHDKIGKGFKTWKISLQEMC**	67-110	43	4867	NIm-IA, NIm-IB
**p4L**	WKISLQEMAQIRRKYELFTYTRFDSEITIVTAAC**	101-133	34	4112	
**p4aL**	AQIRRKYELFTYTRFDSEITIVTAAAAQGDDSGHIVLQFMYVC**	109-150	43	4929	NIm-IB
**p5L**	AAQGDDSGHIVLQFMYVPPGAPVPEKRDDYTWQC**	134-166	34	3792	ICAM-1
**p5aL**	AAAQGDDSGHIVLQFMYVPPGAPVPEKRDDYTWQSGTNASVFC**	133-174	43	4627	ICAM-1
**p6L**	SGTNASVFWQEGQPYPRFTIPFMSIASAYYMFYDC**	167-200	35	4076	
**p7L**	GYDGDSAASKYGSVVTNDMGTICVRIVTSNQKHC**	201-233	34	3577	ICAM-1
**p7aL**	ASAYYMFYDGYDGDSAASKYGSVVTNDMGTICVRIVTSNQKHC**	192-233	43	4690	ICAM-1
**p8L**	DLNIVCRIYHKAKHIKAWCPRPPRAVAYQHTHSC**	234-266	34	4013	
**p9L**	TNYIPSNGEATTQIKTRPDVFTGTNVC**	267-292	27	2928	NIm-IV

**Definition of abbreviations:* S, short; L, long, aa, amino acid.
†Underlined are 5 aa overlapping sequences between peptides.
‡Position denotes the corresponding region of the 89VP1 protein (GenBank accession number: AAQ19944.1).
§Length: number of amino acids, aa.
‖NIm IA, IB, IV, denote binding sites for neutralizing antibodies and ICAM-1, respectively.
**Cysteine residues added for coupling are underlined.

Supplementary Table 5. Structural and non-structural RV proteins and fragments spotted on the PreDicta chip.

Species	Strain	Protein	Expression vector	Length [aa][*]	Calculated molecular weight [kDa][†]	Calculated isoelectric point[†]	GenBank accession number of source strains
		Structural proteins					
**RV-A**	**89**	89VP1	pET27b	1-292	33.6	6.08	AAQ19944.1[‡]
		89VP2	pET27b	1-267	30.6	6.37	AAA45762.1
		89VP3	pET27b	1-238	27.5	6.23	AAA45762.1
		89VP4	pET27b	1-68	8.3	7.06	AAA45762.1
		89VP1-PI	pMALC4X	1-100	54.9	5.15	AAQ19944.1[3]
		89VP1-PII	pMALC4X	101-200	55.7	5.36	AAQ19944.1[3]
		89VP1-PIII	pMALC4X	201-292	54.2	6.22	AAQ19944.1[3]
		89VP2-PI	pMALC4X	1-90	54.0	5.28	AAA45762.1
		89VP2-PII	pMALC4X	91-180	54.0	5.69	AAA45762.1
		89VP2-PIII	pMALC4X	181-267	53.4	5.79	AAA45762.1
		89VP3-PI	pMALC4X	1-80	53.0	5.34	AAA45762.1
		89VP3-PII	pMALC4X	81-160	52.8	5.71	AAA45762.1
		89VP3-PIII	pMALC4X	161-238	52.7	5.54	AAA45762.1
**RV-A**	**16**	16VP1	pET27b	1-285	33.4	6.65	AAQ19871.1
		16VP2	pET27b	1-261	29.9	6.52	Q82122.4
		16VP3	pET27b	1-238	26.3	6.21	Q82122.4
		16VP4	pET27b	1-68	8.31	7.06	Q82122.4
		16VP1-PI	pMALC4X	1-100	55.3	5.06	AAQ19871.1
		16VP1-PII	pMALC4X	101-200	55.6	5.53	AAQ19871.1
		16VP1-PIII	pMALC4X	201-285	53.8	6.98	AAQ19871.1
**RV-A**	**02**	02VP1	pET27b	1-283	33.1	6.34	AAQ19857.1
		02VP2	pET27b	1-261	29.9	6.72	CAA26181.1
		02VP3	pET27b	1-237	27.0	6.81	CAA26181.1
		02VP4	pET27b	1-68	8.3	7.06	CAA26181.1
		02VP1-PI	pMALC4X	1-100	55.1	5.06	AAQ19857.1
		02VP1-PII	pMALC4X	101-200	55.6	5.34	AAQ19857.1
		02VP1-PIII	pMALC4X	201-283	53.7	6.77	AAQ19857.1
**RV-C**	**YP**	YPVP1	pET27b	1-274	31.8	6.92	YP001552435.1
		YPVP2	pET27b	1-261	29.7	6.58	YP001552433.1
		YPVP3	pET27b	1-235	26.8	6.10	YP001552434.1
		YPVP4	pET27b	1-66	8.0	7.06	YP001552432.1
		YPVP1-PI	pMALC4X	1-100	55.0	5.29	YP001552435.1
		YPVP1-PII	pMALC4X	101-200	55.6	5.36	YP001552435.1
		YPVP1-PIII	pMALC4X	201-274	52.7	6.36	YP001552435.1
**RV-B**	**14**	14VP1	pET27b	1-289	33.5	6.76	AAQ19869.1
		14VP2	pET27b	1-262	29.4	6.52	NP_041009.1
		14VP3	pET27b	1-236	27.1	6.23	NP_041009.1
		14VP4	pET27b	1-68	8.1	7.06	NP_041009.1
		14VP1-PI	pMALC4X	1-100	54.9	5.35	AAQ19869.1
		14VP1-PII	pMALC4X	101-200	55.3	5.43	AAQ19869.1
		14VP1-PIII	pMALC4X	201-274	54.7	6.26	AAQ19869.1
		Non-structural proteins					
**RV-A**	**89**	2A *(Protease)*	pET27b	1-136	16.5	5.69	AAA45762.1
		2C *(NTPase)*	pET27b	1-321	37.1	8.87	AAA45762.1
		3A (*unknown*)	pET27b	1-76	9.3	6.25	AAA45762.1
		3C *(Protease)*	pET27b	1-183	21.0	6.62	AAA45762.1
		3D *(Polymerase)*	pET27b	1-460	52.8	6.60	AAA45762.1
		3B *(VPg)*	synthetic peptide	1-21	23.4	10.27	AAA45762.1

Definition of abbreviations: kDa, kilo Dalton, aa, amino acid.
[*]Number of the first and the last aa of the corresponding protein.
[†]Based on the aa sequences, including 1xM (Methionine), 6xH (Histidine) and 1xEF (restriction sites for *EcoRI*: proteins only), calculated with ProtParam, Expasy. [‡]GenBank accession number for 89VP1 is given for the most similar isolate.

Supplementary Table 6. List of allergens and control proteins spotted on the PreDicta chip.

No.	Source	Name	Rec. [R] or natural [N]	IgA/IgG calibration
1	*Peanut*	Ara h 1	R	IgG
2	*Birch*	Bet v 1	R	IgG
3	*Control-protein*	BSA	R	IgG
4	*Dog*	Can f 1	R	IgG
5		Can f 3	N	IgG
6	*Bermuda grass*	Cyn d 1	N	IgG
7	*Wheat Ɣ-gliadins*	Clone 79	R	IgA/IgG
8		Clone 85	R	IgA/IgG
9		Clone 110	R	IgA
10		GG1	R	IgA
11		m43	R	IgG
12	*House dust mite*	Der p 1	N	IgG
13		Der p 10	R	IgG
14	*Egg*	Gal d 1	N	IgG
15	*Olive*	Ole e 9	R	IgA
16	*Human*	HSA	N	IgA/IgG
17	*E. coli*	MBP	R	n.a

Definition of abbreviations: n.a, not applicable.

Supplementary Table 7. Intra- and inter-assay variations among RV microarrays measured on the same day and on 5 consecutive days, respectively.

		INTRA-ASSAY, 6 replicates					INTER-ASSAY, 5 replicates				
**Serum sample**	**ISU-G***	**Antigen, *n***	**%CV**		**SNR**		**Antigen, *n***	**%CV**		**SNR**	
			**Mean**	**SD**	**Mean**	**SD**		**Mean**	**SD**	**Mean**	**SD**
**NS1**	> 50	19	10.04	2.07	10.39	2.28	20	14.01	1.02	7.49	1.85
**1:200**	25 - 50	12	12.55	3.49	8.50	2.16	12	14.42	4.24	7.59	2.61
	5 - 25	19	12.21	4.99	9.27	3.11	19	13.98	5.70	9.37	8.17
	1 - 5	29	26.67	19.75	5.86	3.63	32	28.83	20.82	5.21	2.90
	**Mean**		**15.37**	**7.57**	**8.51**	**2.80**		**17.81**	**7.95**	**7.42**	**3.88**
**NS2**	> 50	8	9.38	2.59	4.47	0.90	9	10.62	2.18	9.70	1.61
**1:200**	25 - 50	12	10.06	1.70	2.36	0.50	11	13.04	3.10	8.06	1.89
	5 - 25	34	14.46	9.91	0.84	0.34	38	17.58	10.45	7.14	3.18
	1 - 5	32	18.72	13.70	0.18	0.07	30	21.56	15.65	6.47	3.28
	**Mean**		**13.16**	**6.98**	**1.96**	**0.45**		**15.70**	**7.84**	**7.84**	**2.49**
**NS3**	> 50	3	10.74	0.79	9.34	0.68	5	13.03	4.81	8.72	3.76
**1:500**	25 - 50	19	9.46	2.86	11.57	3.61	17	17.27	5.64	6.41	2.14
	5 - 25	23	11.15	4.16	10.52	4.54	23	17.16	5.84	6.33	2.00
	1 - 5	31	21.39	12.87	6.85	4.25	34	25.02	13.78	4.99	2.09
	**Mean**		**13.19**	**5.17**	**9.57**	**3.27**		**18.12**	**7.52**	**6.61**	**2.50**
**NS4**	> 50	5	9.88	1.59	10.39	2.06	3	7.68	1.09	13.22	2.02
**1:2000**	25 - 50	16	11.17	3.41	10.01	3.98	17	8.89	4.15	14.00	6.86
	5 - 25	25	13.81	5.37	8.75	5.12	25	13.98	6.49	8.61	4.11
	1 - 5	17	31.66	27.76	5.83	4.26	18	25.90	15.14	4.79	1.86
	**Mean**		**16.63**	**9.53**	**8.74**	**3.85**		**14.11**	**6.72**	**10.16**	**3.71**

Definition of abbreviations: NS, normal serum; CV, coefficient of variation, SNR, signal to noise ratio; SD, standard deviation.
*cut-off: 1 ISU-G.
